# Psychological distress and the perception of radiation risks: the Fukushima health management survey

**DOI:** 10.2471/BLT.14.146498

**Published:** 2015-06-15

**Authors:** Yuriko Suzuki, Hirooki Yabe, Seiji Yasumura, Tetsuya Ohira, Shin-Ichi Niwa, Akira Ohtsuru, Hirobumi Mashiko, Masaharu Maeda, Masafumi Abe, on behalf of the Mental Health Group of the Fukushima health management survey

**Affiliations:** a National Institute of Mental Health, National Center of Neurology and Psychiatry, Department of Adult Mental Health, 4-1-1 Ogawa-Higashi, Kodaira, Tokyo 187-8553, Japan.; b Fukushima Medical University, Fukushima, Japan.; c Fukushima Prefecture Developmental Disability Support Center, Fukushima, Japan.

## Abstract

**Objective:**

To assess relationships between the perception of radiation risks and psychological distress among evacuees from the Fukushima nuclear power plant disaster.

**Methods:**

We analysed cross-sectional data from a survey of evacuees conducted in 2012. Psychological distress was classified as present or absent based on the K6 scale. Respondents recorded their views about the health risks of exposure to ionizing radiation, including immediate, delayed and genetic (inherited) health effects, on a four-point Likert scale. We examined associations between psychological distress and risk perception in logistic regression models. Age, gender, educational attainment, history of mental illness and the consequences of the disaster for employment and living conditions were potential confounders.

**Findings:**

Out of the 180 604 people who received the questionnaire, we included 59 807 responses in our sample. There were 8717 respondents reporting psychological distress. Respondents who believed that radiation exposure was very likely to cause health effects were significantly more likely to be psychologically distressed than other respondents: odds ratio (OR) 1.64 (99.9% confidence interval, CI: 1.42–1.89) for immediate effects; OR: 1.48 (99.9% CI: 1.32–1.67) for delayed effects and OR: 2.17 (99.9% CI: 1.94–2.42) for genetic (inherited) effects. Similar results were obtained after controlling for individual characteristics and disaster-related stressors.

**Conclusion:**

Among evacuees of the Fukushima nuclear disaster, concern about radiation risks was associated with psychological distress.

## Résumé

**Objectif:**

Évaluer les liens entre la perception des risques liés aux rayonnements et la détresse psychologique chez des personnes évacuées suite à la catastrophe survenue à la centrale nucléaire de Fukushima.

**Méthodes:**

Nous avons analysé des données transversales tirées d'une enquête réalisée en 2012 auprès des personnes évacuées. La détresse psychologique a été classée comme présente ou absente en utilisant l'échelle K6. Dans l’enquête, les répondants avaient évalué leur perception des risques sanitaires liés à l'exposition à des rayonnements ionisants -effets sur la santé immédiats, différés et génétiques (hérités)- sur une échelle de Likert à quatre points. Nous avons étudié les associations entre détresse psychologique et perception des risques à l'aide de modèles de régression logistique. L'âge, le sexe, le niveau d'études, les antécédents de maladies mentales et les conséquences de la catastrophe sur l'emploi et les conditions de vie ont été identifiés comme des facteurs de confusion potentiels.

**Résultats:**

180 604 personnes avaient reçu le questionnaire. Dans notre échantillon, nous avons inclus 59 807 réponses. Pour 8 717 personnes, les réponses traduisent la présence d'une détresse psychologique. Les répondants qui estimaient que l'exposition aux rayonnements était très susceptible d'avoir des effets sur la santé ont été largement plus enclins à souffrir de détresse psychologique comparativement aux autres répondants: rapport des cotes (RC) 1,64 (intervalle de confiance (IC) de 99,9%: 1,42–1,89) pour les effets immédiats; RC: 1,48 (IC 99,9%: 1,32–1,67) pour les effets différés et RC: 2,17 (IC 99,9%: 1,94–2,42) pour les effets génétiques (hérités). Des résultats similaires ont été obtenus après contrôle des caractéristiques individuelles et des facteurs de stress liés à la catastrophe.

**Conclusion:**

Chez les personnes évacuées suite à la catastrophe nucléaire de Fukushima, les inquiétudes concernant les risques liés aux rayonnements ont été associées à une détresse psychologique.

## Resumen

**Objetivo:**

Evaluar la relación entre la percepción de los riesgos de radiación y la angustia psicológica entre los evacuados del desastre de la central nuclear de Fukushima.

**Métodos:**

Se analizaron los datos transversales de una encuesta realizada a los evacuados en 2012. La angustia psicológica se clasificó en términos de presente o ausente en base a la escala K6. Los encuestados registraron sus opiniones sobre los riesgos sanitarios de la exposición a la radiación ionizante, incluyendo los efectos inmediatos, tardíos o genéticos (heredados) en la salud en una escala Likert de cuatro puntos. Se examinaron las asociaciones entre la angustia psicológica y la percepción de los riesgos en modelos logísticos de regresión. La edad, el género, el nivel educativo, el historial de enfermedades mentales y las consecuencias del desastre en cuanto a empleo y vida fueron factores potenciales de confusión.

**Resultados:**

En nuestra muestra se incluyeron 59.807 respuestas de las 180.604 personas que realizaron el cuestionario. Hubo 8.717 encuestados que registraron angustia psicológica. Aquellos encuestados que creían que la exposición a la radiación muy probablemente causaría efectos sobre la salud tenían más posibilidades de estar psicológicamente angustiados que otros encuestados: cociente de posibilidades (CP) de 1,64 (intervalo de confianza, IC, del 99,9%: 1,42–1,89) para efectos inmediatos; CP: 1,48 (IC del 99,9%: 1,32-1,67) para efectos tardíos, y CP: 2,17 (IC del 99,9%: 1,94–2,42) para efectos genéticos (heredados). Se obtuvieron resultados parecidos tras controlar las características individuales y los agentes de estrés relacionados con desastres.

**Conclusión:**

En los evacuados del desastre nuclear de Fukushima, la preocupación en relación con los riesgos de radiación estaba asociada con la angustia psicológica.

## ملخص

**الغرض:**

تقييم العلاقات القائمة ما بين إدراك مخاطر الإشعاع والكرب النفسي بين الضحايا الذين تم إجلاؤهم من الكارثة النووية التي أحاقت بمفاعل فوكوشيما النووي.

**الطريقة:**

قمنا بتحليل البيانات المأخوذة من قطاعات متعددة والمستخلصة من مسح شمل الضحايا الذين تم إجلاؤهم كان قد تم إجراؤه في عام 2012. وتم تصنيف الكرب النفسي على أنه حالة قائمة أو غير موجودة بناءً على مقياس K6. وقد سجّل المشاركون وجهات نظرهم بشأن المخاطر الصحية المرتبطة بالتعرض للإشعاع المُؤيِّن، بما يشمل الآثار الصحية الفورية والمؤجلة والجينية (الموروثة) على مقياس ليكرت رباعي النقاط. كما نظرنا في علاقات الاقتران ما بين الكرب النفسي وإدراك الخطر في نماذج التحوف (Regression model) اللوجيستي. وظهرت عوامل السن والجنس والتحصيل التعليمي وتاريخ المرض العقلي وتبعات الكارثة على التوظيف وظروف المعيشة كعوامل مُربكة (Confounders).

**النتائج:**

من بين 180,604 شخصًا تسلموا الاستبيان، فقد قمنا بتضمين 59,807 استجابة في عينتنا. وقد أبلغ 8717 مشاركاً عن الإصابة بكرب نفسي. وكان المشاركون الذين يعتقدون أن التعرض إلى الإشعاع يمثل احتمالاً وارداً للغاية للتسبب في آثار صحية يواجهون احتمالاً أعلى للإصابة بالكرب النفسي بالمقارنة مع غيرهم من المشاركين: بنسبة احتمال 1.64 (بفاصل ثقة 99.9% يبلغ: 1.42‏–1.89) للآثار الفورية، ونسبة احتمال: 1.48 (بنسبة أرجحية مقدارها 99.9%: 1.32‏–1.67) للآثار المؤجلة، ونسبة احتمال: 2.17 (بنسبة أرجحية مقدارها 99.9%: 1.94–2.42) للآثار الجينية (الموروثة). وتم الحصول على نتائج مشابهة بعد التحكم في الخصائص الفردية ومسببات الكرب المرتبطة بالكارثة.

**الاستنتاج:**

كان القلق بشأن مخاطر الإشعاع يرتبط بالكرب النفسي بين الضحايا الذين تم إجراؤها من كارثة فوكوشيما النووية.

## 摘要

**目的:**

旨在评估对辐射风险的认知与经历福岛核电站灾难的撤离人员的心理压力之间的关系。

**方法:**

我们分析了从在  2012 年开展的撤离人员调查中获取的横截面数据。根据 K6 量表，心理压力被分为存在或不存在两种情况。 受访者记录了他们对因电离辐射而造成的健康风险的看法，包括李克特 (Likert) 四分量表中对健康产生的直接性、延缓性和基因性（遗传性）影响。 我们采用逻辑回归模型调查了心理压力和风险认知之间的关联。 年龄、性别、受教育程度、精神病史以及灾难对就业和生活条件产生的后果都是潜在的混杂因素。

**结果:**

从收到调查问卷的 180 604 名人员中，我们在抽样调查中囊括了其中 59 807 个人的回答。 其中有 8717 名受访者表明有心理压力。 认为接触辐射非常有可能引起健康问题的受访者比其他受访者更有可能产生心理压力： 直接性影响的比值比 (OR) 为 1.64（99.9% 置信区间，CI： 1.42–1.89），延缓性影响的比值比 (OR) 为 1.48 (99.9% CI: 1.32-1.67)，基因性（遗传性）影响的比值比 (OR) 为 2.17 (99.9% CI: 1.94–2.42)。 在对个人特征和与灾难相关的压力因素进行控制后，得到相似的结果。

**结论:**

在经历了福岛核电站灾难的撤离人员中，对辐射风险的担忧与心理压力有关。

## Резюме

**Цель:**

Оценить связь между восприятием рисков, связанных с радиацией, и психологическими расстройствами среди лиц, эвакуированных из зоны катастрофы на АЭС «Фукусима».

**Методы:**

Был проведен анализ перекрестных данных анкетирования эвакуированных лиц, которое проводилось в 2012 году. Наличие или отсутствие психологических расстройств классифицировалось по шкале К6. Респонденты выражали свое мнение по вопросу риска для здоровья вследствие воздействия ионизирующей радиации, включая непосредственное воздействие, отсроченное и генетическое (наследственное) воздействие на здоровье, давая оценку по четырехбалльной шкале Лайкерта. Была проанализирована связь между психологическими расстройствами и восприятием риска с помощью регрессионных логистических моделей. В числе возможных факторов влияния на результаты могли быть возраст, пол, уровень образования, наличие психических заболеваний в анамнезе и то, как катастрофа сказалась на трудоустройстве и условиях проживания анкетируемых.

**Результаты:**

В нашу выборку мы включили 59 807 ответов из общего числа 180 604 опрошенных лиц. Среди респондентов 8717 человек сообщали о наличии у них психологических расстройств. Респонденты, которые считали, что воздействие радиации с высокой вероятностью может повлиять на здоровье, испытывали психологическое расстройство в значительно большей мере, нежели другие респонденты: отношение шансов (ОШ) составило 1,64 (при 99,9% доверительном интервале, ДИ: 1,42–1,89) для непосредственного воздействия, ОШ 1,48 (99,9% ДИ: 1,32–1,67) для отсроченного воздействия и ОШ 2,17 (99,9% ДИ: 1,94–2,42) для генетических (унаследованных) дефектов. Сходные результаты были получены после корректировки по индивидуальным характеристикам и стрессовым факторам, связанным с катастрофой.

**Вывод:**

Среди лиц, эвакуированных в связи с ядерной катастрофой на АЭС «Фукусима», озабоченность радиационными рисками ассоциируется с психологическим расстройством.

## Introduction

The Tohoku earthquake in Japan on 11 March 2011 was a triple disaster – earthquake, tsunami and nuclear incident – that had major health effects. The Chernobyl nuclear disaster in the former Soviet Union led to increased mental health problems among residents, which persisted and consequently became a public health problem.^1^^,^^2^ Likewise, a high proportion of the evacuees in Fukushima have experienced psychological distress and traumatic reactions.^3^

The complex nature of the events at Fukushima, comprising both natural and technological disasters, created an additional burden on residents’ mental health.^4^ Previous research has identified factors affecting mental health following a disaster, including female gender, low-socioeconomic status, experience of severe disaster damage, poor social support, physical injuries, history of mental illness or traumatic experience and proximity to the disaster site.^5^^–^^8^ Risk perception is an additional factor affecting mental health following a nuclear disaster.^9^ Risk perception concerns the subjective judgment that people make about the characteristics and severity of risks.

Similar factors can reasonably be expected to have affected Fukushima residents; indeed, research on health workers dispatched after the earthquake revealed that their concerns over radiation exposure adversely affected their mental health status.^10^ Moreover, Japanese people were concerned about the risk of radiation even before the Fukushima disaster, as a result of the atomic bombing of Hiroshima and Nagasaki.^11^

After the Fukushima disaster, the local government launched an extensive health survey to reach evacuees at risk of health problems and to monitor their health status.^12^ Here, we assess whether psychological distress is associated with perceived risks of radiation exposure and disaster-related stressors in people who were evacuated from their homes because of the disaster.

## Methods

### Study design

The Fukushima health management survey was implemented to monitor the long-term health and lifestyle changes of the evacuees following the Fukushima disaster. The present study was conducted as a part of a longitudinal study to monitor the mental health status of evacuees of the Fukushima disaster.^12^

The data reported here are from a baseline cross-sectional survey conducted in 2012, within a year of the disaster. The target population were all residents registered within the government-designated evacuation zone, which included the following municipalities: Hirono-machi, Naraha-machi, Tomioka-machi, Kawauchi-mura, Okuma-machi, Futaba-machi, Namie-machi, Katsurao-mura, Iitate-mura, Minamisoma City, Tamura City and part of Date City in Fukushima prefecture. On January 18, 2012, questionnaires were posted out to evacuees who were at least 15 years old on March 11, 2011 (*n* = 180 604). The questionnaire on mental health and lifestyle was self-administered. Reminders were sent, but no incentives were offered to the residents. The study was approved by the Ethics Committee of Fukushima Medical University and the National Center of Neurology and Psychiatry, Japan.

### Data sources

The outcome variable was non-specific psychological distress as measured by the K6 scale.^13^ This scale, which ranges from zero to 24, asks respondents whether they have experienced six mental health symptoms during the past 30 days. Each question is rated on a five-point Likert scale, with higher scores signifying higher psychological distress. The Japanese version of the K6 score has been validated.^14^ We defined psychological distress as a K6 score ≥ 13.^13^

We measured participants’ beliefs about the potential health effects of radiation exposure^15^ based on their responses to the following questions: (i) What do you think is the likelihood of having immediate health damage (e.g. dying within one month) as a result of your current level of radiation exposure? (ii) What do you think is the likelihood of damage to your health (e.g. cancer onset) in later life as a result of your current level of radiation exposure? (iii) What do you think is the likelihood that the health of your future (i.e. as yet unborn) children and grandchildren will be affected as a result of your current level of radiation exposure? These items were translated into Japanese, then back to English, and modified after discussion with the authors of the questionnaire. Participants were asked to respond to each question using a four-point Likert scale as follows: very unlikely (1), unlikely (2), likely (3) or very likely (4).

We also collected data on individual characteristics including age, gender, educational attainment (elementary school or junior high school, high school, vocational college or junior college, university or graduate school) and history of mental illness. Age was categorized as follows: 15–49 years (reproductive age),^16^ 50–64 years and older than 64 years.

Information on disaster-related stressors was collected from the questionnaire, including: living place (in or out of Fukushima prefecture); living arrangement at the time of the survey (evacuation shelter, temporary housing, rental housing/apartment, relative’s house, own house or other); employment (full-time, part-time or unemployed); loss of employment (yes/no); decrease in income (yes/no); damage to house (no damage, partial damage, partial collapse, partial but extensive collapse or total collapse) and death of someone close (yes/no). To examine the effect of multiple disaster stressors, we created a new variable (the number of stressors) equal to the sum of disaster-related stressors in the highest category. The variable was reclassified into quartiles for inclusion in regression models.

### Statistical analysis

We restricted the analysis to participants who responded to all items on the K6 scale. For the remaining variables, missing data were replaced with their respective reference category.

We examined the distribution of demographic characteristics, disaster-related stressors, perceived risks of radiation exposure and psychological distress using *χ^2^* tests. Associations between perceived risks of radiation exposure and psychological distress were investigated in logistic regression models. We ran models with and without inclusion of individual characteristics and disaster-related stressors. Model 1 included disaster-related stressors as separate variables, while model 2 used our derived variable indicating the number of stressors (classified into quartiles).

In a post-hoc analysis, we explored the individual characteristics and disaster-related stressors associated with rating radiation risks as very likely.

Multicollinearity was assessed using variance inflation factors. All statistical analyses were performed using Stata 13.0 for Windows (StataCorp LP, College Station, United States of America).

## Results

The questionnaire was returned by 73 569 (40.7%) of participants. We excluded 9245 responses that were completed by another family member, 4381 that were missing any values for the K6 score and 136 that were missing values for other variables such as gender and age. This resulted in a final sample of 59 807 (33.1%) responses; 8717 participants were classified as psychologically distressed (14.6%). The distribution of the survey variables by degree of psychological distress is presented in Table 1.

**Table 1:** Individual characteristics and disaster-related stressors in Fukushima evacuees by level of psychological distress, Japan, 2012

Characteristic	No. (%)			*P*^a^
	Overall	K6 < 13	K6 ≥ 13	
**Individual characteristics**				
Sex (*n* = 59 807)				
Males	26 321 (44.0)	23 188 (45.4)	3 133 (35.9)	< 0.001
Females	33 486 (56.0)	27 902 (54.6)	5 584 (64.1)	
Age group (*n* = 59 807)				
15–49 years	22 379 (37.4)	19 255 (37.7)	3 124 (35.8)	< 0.001
50–64 years	19 315 (32.3)	16 441 (32.2)	2 874 (33.0)	
≥ 65 years	18 113 (30.3)	15 394 (30.1)	2 719 (31.2)	
Educational attainment				
Elementary, junior high or high school	42 170 (72.9)	35 819 (72.4)	6 351 (75.6)	< 0.001
Vocational college, junior college or more	15 708 (27.1)	13 654 (27.6)	2 054 (24.4)	
History of mental illness (*n* = 57 859)				
No	54 994 (95.0)	48 111 (96.8)	6 883 (84.2)	< 0.001
Yes	2 865 (5.0)	1 577 (3.2)	1 288 (15.8)	
**Disaster-related stressors**				
House damage (*n* = 56 005)				
Less than partial collapse of the house	47 243 (84.4)	41 006 (85.5)	6 237 (77.7)	< 0.001
Partial collapse and worse	8 762 (15.6)	6 977 (14.5)	1 785 (22.3)	
Bereavement (*n* = 58 666)				
No	47 091 (80.3)	41 128 (81.9)	5 963 (70.5)	< 0.001
Yes	11 575 (19.7)	9 074 (18.1)	2 501 (29.5)	
Living place (*n* = 59 807)				
In Fukushima prefecture	48 110 (80.4)	41 473 (81.2)	6 637 (76.1)	< 0.001
Out of Fukushima prefecture	11 697 (19.6)	9 617 (18.8)	2 080 (23.9)	
Living arrangement at time of survey (*n* = 59 807)				
Own house	17 999 (30.1)	16 299 (31.9)	1 700 (19.5)	< 0.001
Other than own house	41 808 (69.9)	34 791 (68.1)	7 017 (80.5)	
Type of work (*n* = 59 695)				
Full-time	15 934 (26.7)	14 187 (27.8)	1 747 (20.1)	< 0.001
Other than full-time	43 761 (73.3)	36 801 (72.2)	6 960 (79.9)	
Became unemployed (*n* = 59 807)				
No	47 085 (78.7)	40 949 (80.2)	6 136 (70.4)	< 0.001
Yes	12 722 (21.3)	10 141 (19.8)	2 581 (29.6)	
Income has decreased (*n* = 59 807)				
No	48 441 (81.0)	41 690 (81.6)	6 751 (77.4)	< 0.001
Yes	11 366 (19.0)	9 400 (18.4)	1 966 (22.6)	
Number of stressors^b^ (*n* = 59 807)				
0–1	17 390 (29.1)	16 017 (31.4)	1 373 (15.8)	< 0.001
2	16 144 (27.0)	13 943 (27.3)	2 201 (25.2)	
3	14 150 (23.7)	11 723 (22.9)	2 427 (27.8)	
4–7	12 123 (20.3)	9 407 (18.4)	2 716 (31.2)	

^a^ *χ^2^* tests were used.

^b^ Total number of ‘yes’ answers on above seven disaster-related stressors, and severe disaster damage.

Note: Psychological distress was measured using K6 scale.^13^ A score ≥ 13 was defined as psychological distress.

**Fig. 1:**
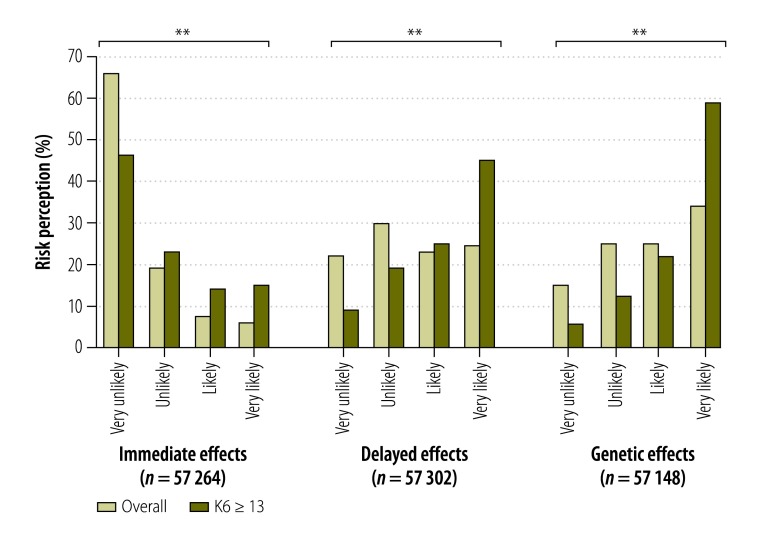
Perception of radiation risks and psychological distress in Fukushima evacuees, Japan, 2012

Fig. 1 summarizes participants’ perception of radiation risks to health. The most frequent responses were as follows: immediate effects were considered very unlikely, delayed effects were unlikely and genetic effects were very likely. Compared to people without psychological distress, more people with psychological distress thought that immediate, delayed and genetic effects were very likely.

** *P* < 0.001.

Note: Psychological distress was measured using K6 scale.^13^ A score ≥ 13 was defined as psychological distress.

In the unadjusted logistic regression analysis, psychological distress was positively associated with the perception that radiation risks were very likely for immediate effects (odds ratios, OR: 1.70; 99.9% confidence interval, CI: 1.48–1.95), for delayed effects (OR: 1.52; 99.9% CI: 1.36–1.70) and for genetic effects (OR: 2.35; 99.9% CI: 2.11–2.62).

In the adjusted models, after controlling for individual characteristics and disaster-related stressors, the corresponding ORs were not changed substantially, OR: 1.64 (99.9% CI: 1.42–1.89) for immediate effects, OR: 1.48 (99.9% CI: 1.32–1.67) for delayed effects and OR: 2.17 (99.9% CI: 1.94–2.42) for genetic effects (Table 2, model 1). The following variables were associated with increased psychological distress: being female, history of mental illness, experience of partial collapse or more severe house damage, experience of bereavement, not living in own house, current working type of other than full-time, loss of employment and decreased income. Higher educational attainment was associated with decreased psychological distress. In model 2, increased psychological distress was associated with the number of disaster-related stressors (Table 2, model 2).

**Table 2:** Individual characteristics, disaster-related stressors, perception of radiation risks and psychological distress in Fukushima evacuees, Japan, 2012 (*n* = 56 556)

Variable	Evacuees with physiological distress,^a^ OR (99.9% CI)	
	Model 1^b^	Model 2^c^
**Perception of radiation risk**		
Immediate effects (reference: less than very likely (1–3))		
Very likely (4)	1.64 (1.42–1.89)	1.64 (1.42–1.89)
Delayed effects (reference: less than very likely (1–3))		
Very likely (4)	1.48 (1.32–1.67)	1.48 (1.32–1.66)
Genetic effects (reference: less than very likely (1–3))		
Very likely (4)	2.17 (1.94–2.42)	2.19 (1.96–)2.44
**Individual characteristics**		
Sex (reference: males)		
Females	1.37 (1.25–1.50)	1.35 (1.23–1.47)
Age group (reference: 15–49 years)		
50–64 years	1.07 (0.97–1.19)	1.08 (0.97–1.19)
≥ 65 years	1.08 (0.96–1.21)	1.05 (0.94–1.17)
Educational attainment (reference: elementary, junior high or high school)		
Vocational college, junior college, or more	0.90 (0.81–0.99)	0.90 (0.81–0.99)
History of mental illness (reference: no)		
Yes	5.00 (4.33–5.77)	4.97 (4.30–5.74)
**Disaster-related stressors**		
House damage (reference: less than partial collapse)		
Partial collapse and worse	1.20 (1.08–1.34)	–
Bereavement (reference: no)		
Yes	1.50 (1.36–1.65)	–
Living place (reference: in Fukushima prefecture)		
Out of Fukushima prefecture	1.05 (0.95–1.17)	–
Living arrangement at time of survey (reference: own house)		
Other than own house	1.55 (1.39–1.73)	–
Type of work (reference: full-time)		
Other than full-time	1.17 (1.04–1.31)	–
Became unemployed (reference: no)		
Yes	1.29 (1.16–1.42)	–
Income has decreased (reference: no)		
Yes	1.21 (1.10–1.35)	–
Number of stressors (reference: 0–1)		
2	–	1.67 (1.47–1.89)
3	–	2.05 (1.81–2.33)
4–7	–	2.66 (2.34–3.01)

CI: confidence interval; OR: odds ratio.

^a^ Psychological distress was measured using K6 scale.^13^ A score ≥ 13 was defined as psychological distress.

^b^ Included disaster-related stressors as separate variables.

^c^ Used our derived variable indicating the number of stressors.

Notes: Questionnaires with full response were included in the analysis, *n* = 56 556.

Characteristics of participants who perceived radiation risks to be very likely are shown in Table 3. The common stressors associated with greater perceived risk were: experience of bereavement, severe housing damage, not owning the place of residence and decreased income, for all three types of health effects. On the other hand, higher educational attainment was associated with lower perceived risk. People aged over 65 years were more concerned about immediate effects, while women, those living outside Fukushima prefecture and those who had lost employment were more concerned about delayed and genetic effects. Respondents in the age group 15–49 years were more concerned about delayed effects, while older age groups (50–64 years and 65 years and older) were more concerned about genetic effects. The variance inflation factors of the variables in each analysis ranged from 1.01 to 1.92, suggesting a low degree of multicollinearity.

**Table 3:** Individual characteristics, disaster-related stressors and the perception that health effects of exposure to radiation are very likely in Fukushima evacuees, Japan, 2012

**Variable**	**OR (99.9% CI)**		
	**Immediate health effects very likely (*n* = 60 132)**	**Delayed health effects very likely (*n* = 60 303)**	**Genetic effects very likely (*n* = 60 220)**
**Individual characteristics**			
Sex (reference: males)			
Females	1.03 (0.92–1.15)	1.20 (1.12–1.28)	1.22 (1.15–1.29)
Age group (reference: 15–49 years)			
50–64 years	1.11 (0.96–1.28)	0.88 (0.82–0.95)	1.12 (1.04–1.20)
≥ 65 years	1.78 (1.53–2.07)	0.98 (0.90–1.07)	1.31 (1.21–1.42)
Educational attainment (reference: elementary, junior high or high school)			
Vocational college, junior college or more	0.67 (0.58–0.77)	0.83 (0.77–0.90)	0.81 (0.76–0.87)
**Disaster-related stressors**			
House damage (reference: less than partial collapse)			
Partial collapse and worse	1.59 (1.39–1.82)	1.28 (1.18–1.40)	1.22 (1.12–1.32)
Bereavement (reference: no)			
Yes	1.46 (1.29–1.65)	1.39 (1.29–1.50)	1.42 (1.32–1.52)
Living place (reference: in Fukushima prefecture)			
Out of Fukushima prefecture	1.03 (0.90–1.19)	1.19 (1.10–1.29)	1.08 (1.00–1.17)
Living arrangement at time of survey (reference: own house)			
Other than own house	1.22 (1.07–1.40)	1.17 (1.08–1.26)	1.14 (1.07–1.22)
Type of work (reference: full-time)			
Other than full-time	1.06 (0.90–1.24)	0.97 (0.89–1.06)	0.98 (0.91–1.06)
Became unemployed (reference: no)			
Yes	1.14 (0.99–1.31)	1.23 (1.13–1.33)	1.26 (1.17–1.36)
Income has decreased (reference: no)			
Yes	1.36 (1.19–1.55)	1.39 (1.29–1.50)	1.39 (1.29–1.49)

CI: confidence interval; OR: odds ratio.

## Discussion

Among evacuees of the Fukushima disaster, psychological distress was more frequent among people who perceived health effects of radiation exposure to be very likely, even after controlling for possible confounders. In terms of risk perception, the result of this study was consistent with findings from studies conducted in Chernobyl, which indicated that greater perceived radiation risks were associated with poor mental health.^9^^,^^17^^,^^18^ Incorrect understanding of health effects of radiation was related to poor mental health status in a study of people in Nagasaki, Japan, who had not been directly exposed to the atomic explosion.^19^Taken together, it appears that psychological status is related to the perception of radiation risks. 

The proportion of those with psychological distress was far greater in our study (14.6%) than in other areas affected by the Tohoku earthquake and subsequent tsunami (6.2%)^20^ or the Japanese population under normal circumstances (4.2–4.4%).^21^ It is estimated that the prevalence of mental health problems may double at times of disaster.^22^ However, the proportion of people with psychological distress was more than double among the evacuees of the Fukushima disaster, compared to the Japanese population under normal conditions. This may have been due to the complex nature of this disaster, which involves uncertainty about the radiation effects on health.

Factors associated with psychological distress reported in previous disaster research^5^^–^^7^ were also associated with psychological distress here, demonstrating that these findings were generally consistent with previous studies of other types of disaster. One exception was that living outside Fukushima prefecture was not associated with psychological distress among the study population. Generally, relocation as a consequence of disaster has either no association or a negative association with mental health status, depending on the type of disaster.^23^ In the event of a complex disaster such as the Fukushima disaster, living in an unfamiliar place might not strongly affect psychological distress, especially for those who voluntarily chose to move away from Fukushima.

There were weak associations between individual disaster-related stressors and psychological distress. However, there were stronger associations with the number of disaster-related stressors. Predictably, those who experienced more severe disaster damage had more subsequent lifestyle changes, such as moving homes, changing jobs and experiencing a decrease in income and these stressors may have been correlated with each other. Nevertheless, the notion of cumulative disaster stressors has practical implications in providing care. By identifying people who experienced more hardship after the disaster, we can identify those who are more likely to experience psychological distress. This is in line with the approach of psychological first aid, which aims to promote the psychosocial well-being of the people affected by a disaster by assessing and offering practical help.^24^

People who had experienced more severe disaster damage were more concerned about radiation risk. This might reflect the participant’s proximity to the nuclear power plant; however, we do not have data to confirm this speculation and further studies are needed.

Elderly people (65 years and older) were more concerned about immediate effects than younger age groups. On the other hand, respondents of reproductive age were more concerned about delayed effects, whereas respondents older than 49 years were more concerned about genetic effects on their progenies. These different age patterns are consistent with the suggestion that parents and grandparents were concerned about radiation health effects on their children.

Previous studies of nuclear disasters and mental health were done long after the disaster,^25^^,^^26^ or were done in specific populations, such as mothers^27^^–^^29^ and clean-up workers.^30^ Our study was conducted within one year of the disaster. Nevertheless, this study does have some limitations, especially low response rate. Another limitation is the use of the K6 scale, which measures non-specific psychological distress. In the context of disasters, the clinical significance of the chosen threshold score of 13 is not clear. However, brief measures of psychological distress such as the K6 are relevant because the evacuees experienced continuous life stressors such as relocation or uncertainty regarding radiation health effects as well as the immediate effects of the earthquake and tsunami. Finally, we can only infer association, not causality, because of the cross-sectional study design.

The results obtained here cannot be generalized to evacuees in other disasters or other populations under normal circumstances, as each disaster has different features and the affected community has different social and demographic characteristics. However, this complex disaster is a rare event and the description of psychological status and its associated stressors serve as an important reference for appropriate preparation and response for future disasters of this kind.
